# Prevalence and risk factors of cytomegalovirus reactivation in critically Ill patients with COVID-19 pneumonia

**DOI:** 10.1371/journal.pone.0303995

**Published:** 2024-05-21

**Authors:** Tanapat Tassaneeyasin, Somnuek Sungkanuparph, Sirawat Srichatrapimuk, Attawit Charoensri, Kanin Thammavaranucupt, Kulapong Jayanama, Suppachok Kirdlarp

**Affiliations:** Chakri Naruebodindra Medical Institute, Faculty of Medicine Ramathibodi Hospital, Mahidol University, Samut Prakan, Thailand; Hamad Medical Corporation, QATAR

## Abstract

**Backgrounds:**

In critically ill patients with COVID-19, secondary infections are potentially life-threatening complications. This study aimed to determine the prevalence, clinical characteristics, and risk factors of CMV reactivation among critically ill immunocompetent patients with COVID-19 pneumonia.

**Methods:**

A retrospective cohort study was conducted among adult patients who were admitted to ICU and screened for quantitative real-time PCR for CMV viral load in a tertiary-care hospital during the third wave of the COVID-19 outbreak in Thailand. Cox regression models were used to identify significant risk factors for developing CMV reactivation.

**Results:**

A total of 185 patients were studied; 133 patients (71.9%) in the non-CMV group and 52 patients (28.1%) in the CMV group. Of all, the mean age was 64.7±13.3 years and 101 patients (54.6%) were males. The CMV group had received a significantly higher median cumulative dose of corticosteroids than the non-CMV group (301 vs 177 mg of dexamethasone, *p*<0.001). Other modalities of treatments for COVID-19 including anti-viral drugs, anti-cytokine drugs and hemoperfusion were not different between the two groups (*p*>0.05). The 90-day mortality rate for all patients was 29.1%, with a significant difference between the CMV group and the non-CMV group (42.3% vs. 24.1%, *p* = 0.014). Median length of stay was longer in the CMV group than non-CMV group (43 vs 24 days, *p*<0.001). The CMV group has detectable CMV DNA load with a median [IQR] of 4,977 [1,365–14,742] IU/mL and 24,570 [3,703–106,642] in plasma and bronchoalveolar fluid, respectively. In multivariate analysis, only a cumulative corticosteroids dose of dexamethasone ≥250 mg (HR = 2.042; 95%CI, 1.130–3.688; *p* = 0.018) was associated with developing CMV reactivation.

**Conclusion:**

In critically ill COVID-19 patients, CMV reactivation is frequent and a high cumulative corticosteroids dose is a significant risk factor for CMV reactivation, which is associated with poor outcomes. Further prospective studies are warranted to determine optimal management.

## Introduction

Coronavirus Disease 2019 (COVID-19) was officially declared a pandemic by the World Health Organization (WHO) in March 2020. Respiratory failure and acute respiratory distress syndrome (ARDS) are life-threatening complications of severe COVID-19 that rapidly enhance healthcare needs for patients requiring oxygen support. Since the beginning of the pandemic in 2019, there are more than 751 million cases of COVID-19 and 6.8 million deaths reported globally as of January 2023 [1]. Secondary infections are also one of the potentially life-threatening complications that should be aware in critically ill patients with COVID-19 [2].

Superinfections and co-infections with COVID-19 have been reported predominantly in critically ill patients in the intensive care unit (ICU) and are associated with poor outcome [3]. Cytomegalovirus (CMV), a member of the herpesvirus family, has infected most people globally and in a high percentage of the Thai population [4]. Primary CMV infection usually occurs during childhood. Although acute CMV infection in the immunocompetent patients could be asymptomatic or mild and self-limiting, it establishes a latent phase that can be reactivated at the latter of one life. Reactivation is the most common mechanism of CMV disease, especially in immunocompromised patients. Previous literatures on CMV reactivation focused on immunocompromised populations such as solid-organ transplants, hematopoietic cell transplants, and acquired immunodeficiency syndrome (AIDS) patients [5, 6]. There is a growing evidence that critically ill immunocompetent or even severe COVID-19 patients can also develop CMV reactivation and CMV disease [7, 8].

A systematic review by Osawa and Singh revealed several decisive risk factors for developing CMV disease in general critically ill patients, including mechanical ventilation, longer duration of ICU stay, and sepsis [9]. However, many risk factors including age, corticosteroid use, and disease severity are still inconsistent across the literature [9–12]. In addition, virus-induced immune dysfunction may be underestimated as a driver of immunopathogenesis in patients with severe COVID-19, which, together with the administration of immunosuppressive drugs for the treatment of cytokine storm, could lead to CMV reactivation [13]. However, data on CMV reactivation in critically ill patients with COVID-19 is limited. Only a few case reports, case series, and small retrospective studies are available in the medical literature [7, 8, 14–16].

Discrimination between CMV pneumonitis and CMV reactivation with other critical conditions is challenging in practice because the diagnosis depends on the quality of the respiratory sample, histopathological interpretation by skillful pathologist, and the variation of diagnostic tests. Besides, the role of bronchoscopy in the COVID-19 era is a matter of debate owing to the tenuous respiratory status of patients and disease transmission to healthcare staff [17]. Moreover, a definite cut-off for CMV DNA load cannot be established, mainly in plasma CMV DNA load [18]. Given the above considerations, this study aimed (1) to determine the prevalence and clinical characteristics of CMV reactivation among critically ill immunocompetent patients with COVID-19 pneumonia; and (2) to identify risk factors of CMV reactivation in critically ill patients with COVID-19 pneumonia.

## Material and methods

### Study population

We retrospectively reviewed consecutive patients in Chakri Naruebodindra Medical Institute, a tertiary referral hospital in Samut Prakan province of Thailand, during the third wave of the COVID-19 outbreak between March and October 2021 in Thailand.

We collected data on clinical characteristics and laboratory findings from a well-recorded hospital database. Patients were included when all of the following conditions were met: (1) more than 15 years of age; (2) evidence of SARS-CoV-2 infection by real-time polymerase chain reaction (PCR) technique; (3) radiographic findings compatible with pneumonia; (4) admitted to ICU and used either invasive or non-invasive ventilation support; and (5) screened for quantitative real-time PCR for CMV viral load at least once during admission.

Immunocompromised patients were excluded from the study. Patients were classified as immunocompromised when they had any of the following: solid organ or hematopoietic cell transplantation, AIDS, chemotherapy for cancer therapy within three months, receiving prednisolone more than 20 mg/day or equivalent (for at least 14 days) or other immunosuppressive agents for an underlying medical condition prior to COVID-19.

The primary outcome of the study was the all-cause mortality of all study patients. Secondary outcomes were length of stay and complications including acute kidney injury, pulmonary barotrauma, pulmonary embolism, acute fibrinous organizing pneumonia (AFOP)/organizing pneumonia (OP), secondary infections (both bacterial and fungal infection) and septic shock.

Ethical approval was granted by the Research Ethics Committee of the Faculty of Medicine Ramathibodi Hospital, Mahidol University, approval No. MURA 2022/36. The committee approved the use of patient data for the publication of this study.

### Definition and diagnosis of CMV pneumonitis

CMV reactivation was defined as DNAemia ≥ 62 IU/ml in plasma using real-time PCR to detect viral nucleic acid [8]. CMV pneumonitis was defined based on clinical symptoms and signs of pneumonia such as new worsening of pulmonary gas exchange, new infiltrates on imaging, and the presence of quantitative CMV DNA load in either plasma or bronchoalveolar lavage fluid (BALF) as described by Ljungman *et al* [5]. Additionally, we routinely tested for sputum staining, culture and serum galactomannan to exclude other possible causes of pneumonia.

### Quantitative CMV DNA load

Either plasma or BALF was initially collected from all enrolled patients upon ICU admission and then weekly after that. Those samples were subjected to nucleic acid extraction and quantified for CMV DNA using RealTime CMV assay (Abbott Molecular Inc., Des Plaines, IL, USA) and reported in international units/mL (1 copies/mL = 1.56 IU/mL).

### Steroid protocol

During third wave of the COVID-19 outbreak in Thailand, we had developed our local protocol for COVID-19 treatment used in our facility. We used dexamethasone 6 mg/day initially in hypoxic COVID-19 patients with oxygen saturation according to RECOERY trial [19]. In patients who progressively developed hypoxia despite of standard dose of dexamethasone, increasingly dose up to dexamethasone 20 mg/day between 3 to 5 days was recommended and decreased dose of 10 mg/day was used for next 3–5 days or until discharged from ICU, according to the CODEX trial [20]. In severely critical patients who had oxygen saturation less than 90% at ambience, received high-flow oxygen nasal cannula, bi-level positive airway pressure (BiPAP), or mechanically ventilation, methylprednisolone 250–500 mg/day for 3 days was considered, according to Edalatifard *et al* study [21]. Tapering doses of steroids was performed every 3–5 days depending on clinical and oxygenation of each patient.

### Data collection

We collected (1) clinical and demographic data (age, gender, and host risk factors); (2) duration of illness before ICU admission and CMV detection afterward; (3) treatment for COVID-19, including anti-viral drugs, corticosteroids, hemoperfusion, and anti-cytokine drugs (tocilizumab, tofacitinib, and baricitinib); (4) a requirement of invasive mechanical ventilation (IMV) and non-invasive ventilation (NIV); (5) quantitative CMV test results (plasma and BALF CMV DNA load) in all patients; and (6) the sequential organ failure assessment (SOFA) score at the date of ICU admission which were calculated with the parameters including mean arterial pressure or administration of vasopressors required, PaO_2_/FiO_2_ ratio, platelets count, bilirubin and creatinine [22].

### Statistical analyses

Clinical characteristics of critically ill patients with COVID-19 pneumonia and CMV reactivation were analyzed by descriptive analysis. Categorical variables were presented as frequencies and percentages. Continuous variables were presented as mean ± standard deviation (SD) or median [interquartile ranges, IQR]. Chi-square test, Student *t*-test and Mann-Whitney *U* test were used to compare variables between patients with or without CMV reactivation, where appropriate. Univariate and multivariate Cox regression models were used to identify independent risk factors for developing CMV reactivation. Variables that presented *P*-value <0.2, were considered in a multivariate Cox regression model. Hazard ratio (HR) with a 95% confidence interval (CI) were calculated. A *P*-value of <0.05 was considered statistically significant. Additionally, Kaplan–Meier analysis was used for the time-to-event analysis and generating the figure of cumulative probability of developing CMV reactivation. Log-rank test was applied to compare the probability between the two groups of patients. Statistical analysis was performed using Statistical Package for Social Sciences (SPSS) software (version 22, SPSS, Inc, Chicago, IL, USA).

## Results

A total of 185 patients who met the inclusion criteria were studied. Of these, 133 patients (71.9%) were in the non-CMV group and 52 patients (28.1%) in the CMV group. Of all, the mean age was 64.7±13.3 years and 101 patients (54.6%) were males. The mean body mass index (BMI) was higher in the non-CMV group than in the CMV group (24 kg/m^2^ vs. 22 kg/m^2^; *p* = 0.004). The proportion of patients with COPD/asthma was lower in the non-CMV group than in the CMV group (5.3% vs. 15.4%, *p* = 0.023), whereas other comorbidities including diabetes mellitus, coronary artery disease, cerebrovascular disease, and chronic kidney disease were not different between the two groups (*p* > 0.05). The results of laboratory investigations including C-reactive protein, lymphocyte count and lactate dehydrogenase at ICU admission were similar between the two groups (*p* > 0.05). The median [IQR] SOFA score was 3 [2–7] in both groups and there was no significant difference (*p* = 0.355). The CMV group had received a significantly higher median cumulative dose of corticosteroids than the non-CMV group (301 vs 177 mg of dexamethasone, *p* < 0.001). Other modalities of treatments for COVID-19 including anti-viral drugs, anti-cytokine drugs and hemoperfusion were not different between the two groups (*p* > 0.05). All baseline demographic data and characteristics of patients are summarized in **Table 1**.

**Table 1:** Baseline characteristics of patients with COVID-19 pneumonia.

Characteristics	Total *(n = 185)*	CMV group *(n = 52)*	Non-CMV group *(n = 133)*	*P* value
Age (year), mean ±SD	64.7±13.27	67.2±14.9	63.7±12.5	0.107
Male, number (%)	101 (54.6)	32 (61.5)	69 (51.9)	0.236
BMI (kg/m^2^), median [IQR]	24 [21.5–27.0]	22 [19–25]	24 [22.0–27.0]	0.004
BMI <18.5 (kg/m^2^), number (%)	15 (8.1)	12 (23.1)	3 (2.3)	<0.001
BMI 18.5–22.9 (kg/m^2^), number (%)	58 (31.4)	16 (30.8)	42 (31.6)	0.915
BMI 23–24.9 (kg/m^2^), number (%)	32 (17.3)	8 (15.4)	24 (18.0)	0.667
BMI >25 (kg/m^2^), number (%)	80 (43.2)	16 (30.8)	64 (48.1)	0.032
Co-morbidities, number (%)				
Diabetes mellitus	78 (42.2)	23 (44.2)	55 (41.4)	0.722
Coronary artery disease	10 (5.4)	3 (5.8)	7 (5.3)	0.891
COPD/Asthma	15 (8.1)	8 (15.4)	7 (5.3)	0.023
Cerebrovascular disease	11 (5.9)	5 (9.6)	6 (4.5)	0.187
Chronic kidney disease	23 (12.4)	7 (13.5)	16 (12)	0.791
Laboratory parameters at ICU admission, median [IQR]				
Lymphocyte count, (/μL)	834 [579–1108]	774.5 [497–1246]	862 [638–1087]	0.404
C-reactive protein, (mg/L)	53 [25–109]	47.3 [23–109]	59 [25–109]	0.510
Lactate dehydrogenase, (U/L)	372 [290–494]	354 [263–460]	374 [298–497]	0.365
Severity of COVID-19 pneumonia				
SOFA score, median [IQR]	3 [2–7]	3 [2.25–7]	3 [2–7]	0.355
SOFA score category, number (%)				0.378
<5	116 (62.7)	30 (57.7)	86 (64.7)	
≥5	69 (37.3)	22 (42.3)	47 (35.3)	
Treatments, number (%)				
Favipiravir	170 (91.9)	49 (94.2)	121 (91)	0.466
Remdesivir	55 (29.7)	17 (32.7)	38 (28.6)	0.581
Corticosteroids	185 (100)	52 (100)	133 (100)	-
IVMP	151 (81.6)	43 (82.7)	108 (81.2)	0.814
Hemoperfusion	18 (9.7)	8 (15.4)	10 (7.5)	0.105
Invasive mechanical ventilation	108 (58.4)	36 (69.2)	72 (54.1)	0.061
Non-invasive mechanical ventilation	172 (93.0)	47 (90.4)	125 (94)	0.389
Anti-cytokine drugs, number (%)	90 (48.6)	31 (59.6)	59 (44.4)	0.062
Baricitinib	23 (12.4)	8 (15.4)	15 (11.3)	0.447
Tofacitinib	18 (9.7)	6 (11.5)	12 (9)	0.604
Tocilizumab	53 (28.6)	19 (36.5)	34 (25.6)	0.138
Cumulative corticosteroids dose (mg of dexamethasone), median [IQR]	195 [150–275]	301 [225–394]	177 [141–226]	<0.001
IVMP dose (mg of IVMP), median [IQR]	1000 [750–1500]	1125 [750–1500]	1000 [750–1500]	0.263

Abbreviation: CMV = cytomegalovirus; BMI = body mass index; COPD = chronic obstructive pulmonary disease; ICU = intensive care unit; IVMP = intravenous methylprednisolone

The outcomes of all patients are presented in **Table 2**. The 90-day mortality rate for all patients was 29.1%, with a significant difference between the CMV group and the non-CMV group (42.3% vs. 24.1%, *p* = 0.014). The 60-day mortality rate was 28.1%, and it was higher in the CMV group compared to the non-CMV group (38.5% vs. 24.1%, *p* = 0.050). As for the 28-day mortality rate, it was 17.3%, and it was significantly lower in the CMV group than in the non-CMV group (7.7% vs. 21.1%, *p* = 0.031). Median length of stay was longer in the CMV group than non-CMV group (43 vs 24 days, *p*<0.001). The CMV group has detectable CMV DNA load with a median [IQR] of 4,977 [1,365–14,742] IU/mL and 24,570 [3,703–106,642] in plasma and BALF, respectively.

**Table 2:** Outcomes of patients with COVID-19 pneumonia.

Characteristics	Total *(n = 185)*	CMV group *(n = 52)*	Non-CMV group *(n = 133)*	*P* value
***Primary outcome***				
All-cause mortality, number (%)				
28-day mortality	32 (17.3)	4 (7.7)	28 (21.1)	0.031
60-day mortality	52 (28.1)	20 (38.5)	32 (24.1)	0.050
90-day mortality	54 (29.1)	22 (42.3)	32 (24.1)	0.014
***Secondary outcomes***				
Length of stay (days), median [IQR]	28 [21–41]	43 [36–58.7]	24 [19–31.5]	<0.001
Complications, number (%)				
Acute kidney injury	44 (23.8)	21 (40.4)	23 (17.3)	<0.001
Pulmonary barotrauma	23 (12.4)	13 (25)	10 (7.5)	<0.001
Pulmonary embolism	35 (18.9)	19 (36.5)	16 (12.0)	<0.001
AFOP/OP	54 (29.2)	30 (57.7)	24 (18.0)	<0.001
Bacterial infection	92 (49.7)	36 (69.2)	56 (42.1)	<0.001
Fungal infection	22 (11.9)	14 (26.9)	8 (6.0)	<0.001
Septic shock	12 (6.5)	6 (11.5)	6 (4.5)	0.081

Abbreviation: CMV = cytomegalovirus; ICU = intensive care unit; IVMP = intravenous methylprednisolone; AFOP = acute fibrinous organizing pneumonia; OP = organizing pneumonia

In univariate analysis, a cumulative corticosteroids dose of dexamethasone ≥250 mg was associated with CMV reactivation (HR = 2.130; 95% CI, 1.192–3.808; *p* = 0.011) while a C-reactive protein level at ICU admission ≥100 mg/L had a trend toward higher risk for developing CMV reactivation (HR = 1.749; 95% CI, 0.932–3.284; *p* = 0.082). SOFA score ≥5 was not associated with CMV reactivation (HR = 1.342; 95% CI, 0.697–2.583; *p* = 0.379). In multivariate analysis, only a cumulative corticosteroids dose of dexamethasone ≥250 mg (HR = 2.042; 95%CI, 1.130–3.688; *p* = 0.018) was associated with developing CMV reactivation **(Table 3)**.

**Table 3:** Risk factors of CMV reactivation in patients with COVID-19 pneumonia, using univariate and multivariate Cox regression analysis.

Factors	Univariate analysis			Multivariate analysis		
	HR	95% CI	*P* value	HR	95% CI	*P* value
Age (years)	1.009	0.989–1.029	0.394	-	-	-
Male	1.332	0.759–2.339	0.318	-	-	-
BMI ≥23 kg/m^2^	0.639	0.37–1.105	0.109	0.665	0.375–1.179	0.163
Diabetes mellitus	0.820	0.469–1.431	0.484	-	-	-
COPD/Asthma	1.232	0.575–2.640	0.592	-	-	-
Coronary artery disease	0.729	0.226–2.353	0.597	-	-	-
Cerebrovascular disease	1.682	0.666–4.251	0.271	-	-	-
Chronic kidney disease	0.884	0.393–1.986	0.765	-	-	-
Lymphocyte count at ICU admission (x10^3^/μL)	1.011	0.089–1.036	0.489	-	-	-
C-reactive protein at ICU admission ≥ 100 mg/L	1.749	0.932–3.284	0.082	1.714	0.854–3.442	0.130
Lactate dehydrogenase at ICU admission	1.000	0.999–1.002	0.553	-	-	-
SOFA score ≥5	1.342	0.697–2.583	0.379	-	-	-
Hospitalization ≥ 4 weeks	2.261	0.638–8.013	0.206	-	-	-
Favipiravir	1.444	0.448–4.652	0.538	-	-	-
Remdesivir	1.343	0.750–2.405	0.321	-	-	-
IVMP	1.609	0.780–3.322	0.198	-	-	-
Cumulative corticosteroids dose of dexamethasone ≥ 250 mg	2.130	1.192–3.808	0.011	2.042	1.130–3.688	0.018
Anti-cytokine drugs	1.565	0.897–2.729	0.114	1.729	0.958–3.120	0.069
Hemoperfusion	1.857	0.858–4.016	0.116	1.459	0.621–3.428	0.386
Invasive mechanical ventilation	0.880	0.482–1.605	0.677	-	-	-

Abbreviation: CMV = cytomegalovirus; BMI = body mass index; COPD = chronic obstructive pulmonary disease; ICU = intensive care unit; IVMP = intravenous methylprednisolone

Ganciclovir was commenced in 30 patients (57.7%), and the median [IQR] of plasma CMV DNA was 6920 [3,976–13,140] IU/mL when ganciclovir was initiated. The median [IQR] time between CMV reactivation to initiation of ganciclovir was 3.5 [3–6] days. Rates of patients with acute kidney injury, pulmonary barotrauma, pulmonary embolism, organizing pneumonia (OP), acute fibrinous organizing pneumonia (AFOP), bacterial superinfection, and fungal superinfection were significantly higher in the CMV group, compared to the non-CMV group (*p*<0.001), as shown in **Table 2**. Over 90% of these complications occurred after the diagnosis of CMV reactivation.

**Fig 1:**
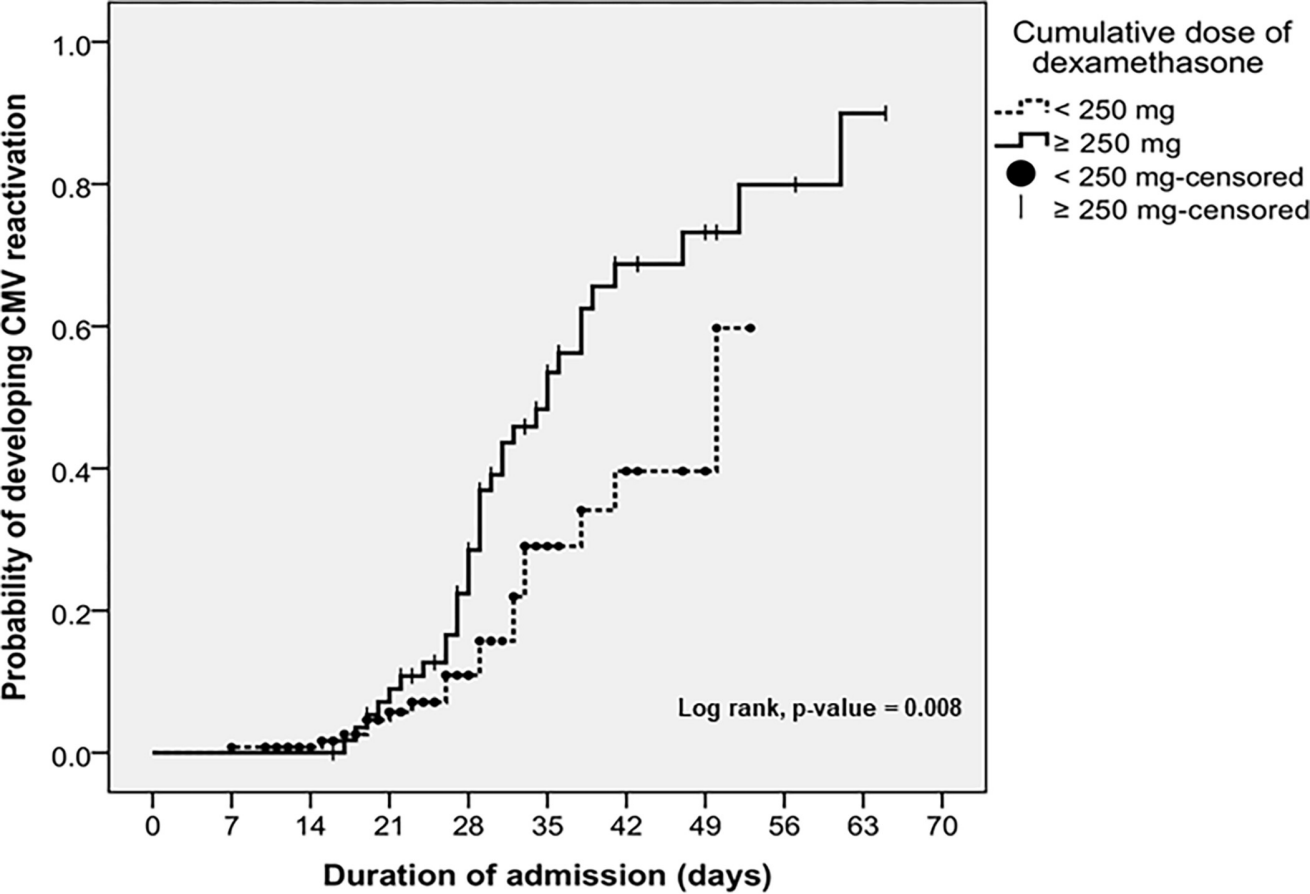
Cumulative probability of developing CMV reactivation in patients with COVID-19 pneumonia.

The cumulative probability of developing CMV reactivation is shown in **Fig 1**. The Kaplan-Meier analysis indicated that cumulative probability of developing CMV reactivation was higher in patients with cumulative corticosteroids dose of dexamethasone ≥ 250 mg (log-rank test, *p* = 0.008). The cumulative incidence of developing CMV reactivation was not significantly different between patients with C-reactive protein at ICU admission ≥ 100 mg/L and < 100 mg/L (log-rank test, *p* = 0.075), as well as patients treated with or without anti-cytokine drugs (log-rank test, *p* = 0.107).

## Discussion

This retrospective cohort study showed that CMV reactivation rate among critically ill patients with COVID-19 pneumonia was high (28.1%). CMV reactivation was associated with several complications, including other secondary infections, pulmonary barotrauma, pulmonary embolism, renal impairment, and AFOP/OP. The occurrence of CMV reactivation was positively associated with all-cause mortality, especially late mortality. The high cumulative dose of corticosteroids increases the risk of CMV reactivation and developing CMV disease. Nevertheless, we did not find an association between anti-cytokine drugs and the risk of developing CMV reactivation.

This study showed that COVID-19 pneumonia patients with CMV reactivation had a higher mortality rate than non-CMV patients. The finding is different from a previous observational study in COVID-19 patients by Gatto *et al* [8], which demonstrated a higher hospital mortality in patients with CMV reactivation but not statistically significant. The factor that may contribute to the different results is the use of high-dose corticosteroids in our study. However, the 28-day mortality was higher in patients without CMV reactivation. It may be explained by the findings of previous studies in immunocompetent critically ill non-COVID-19 patients that it took some period of time to develop CMV reactivation or disease in a critical care setting [9]. Following our study, the median time interval between ICU admission and CMV DNA detection was 26 days. Furthermore, many patients in the non-CMV group did not survive long enough to develop CMV reactivation or disease because of the acute course and other complications of severe COVID-19 pneumonia such as ARDS. Nonetheless, CMV reactivation led to clinical deterioration and higher mortality rates in COVID-19 patients with a longer course of hospitalization. Additionally, secondary bacterial or fungal infections also contribute to the late deterioration [12]. Superimposed infection was observed in severe COVID-19 patients who received mechanical ventilatory support. Use of corticosteroids is one of the strongly and independently associated factor [23].

In the context of critically ill COVID-19, early data suggest immunosuppressant therapies as a treatment for dysregulated inflammatory responses which is contributed to the COVID-19 severity. The RECOVERY trial provides evidence that systemic corticosteroids therapy decrease mortality in COVID-19 patients who are required oxygenation support [19]. Although various anti-cytokine drugs which act against interleukin (IL) -6, Janus kinase 1 and 2 have been proved to be effective against severe COVID-19 [24–29], these were not available at the time of study period.

Several corticosteroid regimens for COVID-19 have been proposed [20, 30]. Many patients in our study received a regimen from the CoDEX trial [20], which has a higher dose of corticosteroids than the RECOVERY trial, because of the progressive, persistent, or relapse of respiratory failure despite dexamethasone regimen as in the RECOVERY trial was administered. Moreover, patients who developed AFOP/OP were commonly treated with corticosteroids. These explain why our patients received a prolonged and very high cumulative dose of corticosteroids. Accordingly, our findings indicated that a higher cumulative dose of corticosteroids increases the risk of CMV reactivation or disease that might negatively affect the prognosis in our patients. As in our cohort, the previous retrospective observational study reported that prolonged corticosteroid therapy was associated with CMV infection in patients with severe COVID-19. Schoninger *et al* found that time from ICU admission to CMV reactivation in COVID-19 patients was 27 days and the median dexamethasone dose was 254 mg [31], which is quite resemble to the results from our study.

The results of the present study strengthen the idea that COVID-19 patients might be in some way immunocompromised, and combined with these effects of corticosteroids therapy might impact CMV reactivation or disease. Therefore, corticosteroids administration should be strictly used only in COVID-19 patients who have the evidence of hypoxemia that need oxygen support according to RECOVERY trial [19] and international treatment guidelines of COVID-19 [32, 33]. Prolonged high-dose corticosteroids should be considered only in selected patients, for example, the COVID-19 patients complicated with AFOP. Moreover, corticosteroids therapy should be discontinued as soon as the resolution of the indication.

Our strengths of this study were that it operated in a center that was fully adapted for the COVID-19 outbreak in Thailand and provided the same standard treatment protocol in all hospitalized patients and the larger sample size when compared to the previous studies. The results have revealed an association between late CMV reactivation and the accumulative use of corticosteroids therapy in the context of COVID-19 treatment which is rarely described. However, some limitations need to be considered. First, due to a single center study, potential limitations in generalization the results to other settings may occur. Second, it was difficult to completely differentiate CMV pneumonitis from other causes of pneumonia with CMV reactivation without BALF or transbronchial lung biopsy since bronchoscopy could not be performed in all cases. Therefore, the diagnosis of possible CMV disease was dependent on physician’s decision-making. Third, we could not conclude whether CMV reactivation and CMV disease were the causative factor of poor outcome in COVID-19 or CMV reactivation only reflected the level of immunosuppressed state from corticosteroid use. Lastly, due to the retrospective study design and limited laboratory access during the outbreak, we did not routinely perform arterial blood gas, which is one of the parameters for SOFA score. We had to use the pulse oximetry (SpO_2_) for calculation of the partial pressures of arterial oxygen (PaO_2_) and PaO_2_/FiO_2_ ratio, respectively. The lack of association between CMV reactivation and other immunosuppressive drugs may be related to inadequate power of study. Future studies should validate our findings by prospective design with the definition of CMV disease.

## Conclusions

In critically ill COVID-19 patients, CMV reactivation is frequent and a high cumulative corticosteroids dose is a significant risk factor for CMV reactivation, which is associated with poor outcomes. Further studies are warranted to determine the appropriate strategy for CMV monitoring and the role of preemptive treatment in COVID-19 patients receiving high dose corticosteroids therapy.

## Supporting information

S1 Table All relevant data of critically ill patients with COVID-19 pneumonia. (XLSX)

## List of abbreviations

AFOP: acute fibrinous organizing pneumonia
AIDS: acquired immune deficiency syndrome
ARDS: acute respiratory distress syndrome
BALF: bronchoalveolar lavage fluid
BMI: body mass index
CI: confident interval
CMV: Cytomegalovirus
COVID-19: Coronavirus Disease 2019
HRs: hazard ratios
ICU: intensive care unit
IL: interleukin
IMV: invasive mechanical ventilation
IQR: interquartile range
IVMP: intravenous pulse methylprednisolone
NIV: non-invasive ventilation
OP: organizing pneumonia
SD: standard deviation
WHO: World Health Organization
